# Neurobiology of Parkinson’s Disease

**DOI:** 10.3390/ijms24129933

**Published:** 2023-06-09

**Authors:** Micaela Morelli, Annalisa Pinna

**Affiliations:** 1 Department of Biomedical Sciences, Section of Neuroscience, University of Cagliari, Monserrato, 09042 Cagliari, Italy; morelli@unica.it; 2 National Research Council of Italy (CNR), Neuroscience Institute—Cagliari, Cittadella Universitaria, Monserrato, 09042 Cagliari, Italy

Parkinson’s disease (PD) is one of the most rapidly growing neurological disorders. PD is characterized by motor deficits preceded by non-motor peripheral dysfunctions and by the presence and progressive propagation of the alpha-synuclein (α-syn) protein and loss of dopaminergic neurons. Moreover, and in addition to the findings linking PD to oxidative stress and neuroinflammation, it should be emphasized that several genes are linked to both familial and autosomal dominant and recessive forms of PD. 

On these bases, this Special Issue aims to understand central and peripheral mechanisms contributing to PD and to explore potential strategies for recovering brain functions from this neurological disorder. In this Special Issue, fourteen papers are published, including original and review articles.

In relation to gene involvement, the Special Issue contains a comprehensive review by Franco et al. [1] describing genes (SNCA, LRRK2, GBA, UCHL1, VPS35, PRKN, PINK1, ATP13A2, PLA2G6, DNAJC6, SYNJ1, DJ-1/PARK7, and FBXO7) involved in familial cases of PD. The review explores their usefulness in deciphering the origin of dopaminergic denervation and describes direct or functional interactions among genes, proposing a map of the interactions between SNCA, the gene encoding for α-syn that aggregates in PD, and other genes. Combining in silico, in vitro, and in vivo data in transgenic animals, two likely mechanisms are described: (a) the processing of native α-syn is altered due to the mutation of genes involved in vesicular trafficking and protein processing, and (b) α-syn mutants alter the mechanisms necessary for correct vesicular trafficking and protein processing. Both mechanisms involve mitochondria and require energy production. On the same topic, the paper by Ogata et al. [2] shows that a novel LRRK2 variant, the p. G2294R in the WD40 domain, has been described in familial PD. Patients carrying the mutation showed late-onset of PD, a good response to levodopa, without cognitive decline or psychosis. Interestingly, this is an example wherein a decrease in LRRK2 activity did not act in a neuroprotective manner. The function of LRRK2 in the immune cells under inflammatory conditions has been further investigated by Oun et al. [3]. This in vitro study demonstrated that stimulation with the inflammatory agent lipopolysaccharide (LPS) enhanced the kinase activity of LRRK2 in parental RAW 264.7 (wild-type: WT) cells and altered cell morphology in LRRK2 deletion (KO) RAW 264.7 cells. Moreover, LRRK2 deletion led to a reduction in interleukin-6 (IL-6) inflammatory cytokine and cyclooxygenase-2 (COX-2) expression and an increase in lactate production after LPS stimulation compared to the WT cells. These findings suggest LRRK2 plays a complex role in inflammatory conditions in peripheral macrophages, and this could affect PD pathology. These data might help the identification of targets that modulate neuroinflammation in PD through LRRK2 function. In addition, the paper by Sucunza et al. [4] shows that bilaterally injected mice or nonhuman primates, with a viral vector coding for the mutated form of α-syn (rAAV9-SynA53T), have reduced α-syn burden and an improved survival of dopaminergic neurons when administered with a vector coding for the GBA1 gene (rAAV9-GBA1) delivered in the *substantia nigra pars-compacta* one month after the initial insult. These results strongly support the important role of GBA1 gene coding for glucocerebrosidase (GCase) as the main genetic risk factor for PD. 

Peripheral deficits, as olfactory and gustatory deficits, and modifications in microbiota have been associated with a large number of patients affected by PD. Deficits in olfaction and taste are among the most frequent non-motor manifestations that start very early and frequently precede the PD motor symptoms. The review by Melis et al. [5] describes the most relevant molecular and genetic factors involved in the PD-related smell and taste impairments, and their associations with the microbiota, describing how several genes controlling different functions are actually involved in the manifestation of this disease. In relation to peripheral influence and α-syn, an interesting aspect took into consideration by the literature on PD is the possibility that the disease has a prion-like characteristic since a-syn spreads from the intestine, through the vagus nerve, to the brain. In the review by Vascellari and Manzin [6], the main characteristic shared between α-syn and prion protein are compared, and the cofactors that influence the remodeling of native protein structures are discussed. Moreover, this review describes the co-factors involved in the *misfolding* process and how in recent decades, scientists have introduced the revolutionary concept that the gut microbiota can influence the function and behavior of the host’s brain by establishing a bidirectional communication, known as the gut–brain axis. It is also emphasized how evidence emerging from animal models of PD suggest that the gut microbiota may promote both neuroinflammation, a common feature of PD, and α-syn aggregation, impairing motor function through the activation of microglia. In relation to α-syn, the neuropathological hallmark of PD, Bluhm et al. [7] describe how recent evidence indicates that oxidative stress induced by metal ions and post-translational modifications, such as phosphorylation, ubiquitination, nitration, glycation, and SUMOylation, affect α-syn conformation. Moreover, they describe proteolytical α-syn cleavage by neurosin, calpain-1, cathepsin D, and matrix metalloproteinase-3 in health and disease. By focusing on oxidative stress, altered intracellular Ca^2+^-homeostasis, mitochondrial dysfunction, and the disruption of mitochondrial integrity. Di Martino et al. [8] have described, in an original paper, the mechanisms leading to mitochondrial dysfunction and its relationship with the activation of the neuroinflammatory process in mice carrying the human mutation of α-syn A53T. In this PD model, they demonstrated that in A53T mice, mitochondrial dysfunction occurs early in midbrain and later in striatum and is mediated by the impairment of NCX3 protein expression in the neurons and astrocytes of the midbrain. Finally, they showed that mitochondrial dysfunction in the midbrain triggers neuroinflammation later into striatum, thus contributing to PD progression during mice aging. 

In the Special Issue, a new tool to study Parkinsonism is also described in the context of aging using the rodent Octodon degus, which is a natural model of multimorbidity. Cuenca-Bermejo et al. [9] describe how MPTP intoxication affects Octodon degus, an attractive natural model for Alzheimer’s disease and other human age-related features, and reports how this rodent is sensitive to MPTP intoxication and could, therefore, be considered a suitable model for experimental Parkinsonism in the context of aging. 

In the area of precision medicine, the manuscript by Kim and Chang [10] describes the management of various adverse effects induced by dopaminergic agents with magnetic nanoparticles. The magnetic nanoparticles, based on human adipose-derived stem cells (hASCs), are utilized in a 6-hydroxydopamine (6-OHDA)-induced PD mouse model. Interestingly, magnetic resonance imaging (MRI) for in vivo tracking revealed that hASCs were distributed in the *substantia nigra* of PD mice, and this was associated with motor behavioral recovery, suggesting a new potential therapeutic strategy in PD. In the same area, we also present a paper by Fajardo-Serrano et al. [11] that provides a review of the current literature, dealing with the use of adeno-associated viruses viral vectors (AAVs), for disease modeling and for therapeutic use purposes. The review summarizes insights on which would be the most appropriate AAV delivery route and on the use of AAVs for modeling synucleinopathies or describing ongoing pre- and clinical initiatives, pushing forward AAV-based therapeutic approaches for PD.

In the neuroimaging diagnostic area, Bonilauri and coworkers [12] utilized the ecological neuroimaging technique “functional near-infrared spectroscopy” (fNIRS) to investigate the cortical activity of PD patients during a motor grasping task and its relationship with both the staging of the pathology and its clinical variables. The results obtained in this study suggest that non-motor areas, principally the prefrontal cortex area, provide a compensation mechanism for PD motor impairment. Thus, the fNIRS provides a non-invasive and ecological neurophysiological correlation of cortical activity. fNIRS, if used in conjunction with current clinical outcomes, may provide a new prodromal, low-cost, and sustainable support for monitoring disease progression (motor and non-motor impairments) and a longitudinal assessment of therapeutic and rehabilitation procedures. 

In the field of non-pharmacologic therapy of PD, several studies have demonstrated that aerobic exercise is an effective strategy for enhancing cognitive function and brain plasticity in PD. In this regard, Tang and collaborators [13] investigated the role of Fibronectin Type III domain containing 5 (FNDC5), a myokine secreted during aerobic exercise, in mediating hippocampal plasticity and regional dopaminergic projections in a progressive model of PD characterized by mice sub-chronically treated with MPTP plus probenecid. The results showed that treadmill exercise alleviated motor dysfunction, cognition disorder, and dopaminergic neuron degeneration induced by MPTP by enhancing FNDC5 levels [13]. Specifically, FNDC5 seems to have a direct effect by promoting the secretion of BDNF in hippocampal neurons and an indirect effect by promoting the dopaminergic connection from the *substantia nigra* to the hippocampus by an interaction between the hippocampal integrin αVβ5 and CD90 on the membrane of dopaminergic terminals. 

Finally, a comprehensive review by Serra et al. [14] describes the role of the protein Rhes (Ras Homolog Enriched in the Striatum) that acts as a modulator of dopamine neurotransmission and is associated with dopaminergic neurons’ degeneration. Rhes transcript is present in several dopamine-related brain areas, and dopamine depletion significantly reduces Rhes mRNA levels in rodents, non-human primates, and PD patients. Rhes modulates levodopa-induced dyskinesia in PD rodent models and is involved in the survival of dopaminergic neurons in rodents, pointing to an involvement of autophagy and mitophagy in these processes.

## References

[1] Franco R., Rivas-Santisteban R., Navarro G., Pinna A., Reyes-Resina I. (2021). Genes Implicated in Familial Parkinson’s Disease Provide a Dual Picture of Nigral Dopaminergic Neurodegeneration with Mitochondria Taking Center Stage. Int. J. Mol. Sci..

[2] Ogata J., Hirao K., Nishioka K., Hayashida A., Li Y., Yoshino N., Shimizu S., Hattori N., Imai Y. (2021). A Novel LRRK2 Variant p.G2294R in the WD40 Domain Identified in Familial Parkinson’s Disease Affects LRRK2 Protein Levels. Int. J. Mol. Sci..

[3] Oun A., Hoeksema E., Soliman A., Brouwer F., García-Reyes F., Pots H., Trombetta-Lima M., Kortholt A., Dolga A.M. (2023). Characterization of Lipopolysaccharide Effects on LRRK2 Signaling in RAW Macrophages. Int. J. Mol. Sci..

[4] Sucunza D., Rico A.J., Roda E., Collantes M., González-Aseguinolaza G., Rodríguez-Pérez A.I., Peñuelas I., Vázquez A., Labandeira-García J.L., Broccoli V. (2021). Glucocerebrosidase Gene Therapy Induces Alpha-Synuclein Clearance and Neuroprotection of Midbrain Dopaminergic Neurons in Mice and Macaques. Int. J. Mol. Sci..

[5] Melis M., Haehner A., Mastinu M., Hummel T., Tomassini Barbarossa I. (2021). Molecular and Genetic Factors Involved in Olfactory and Gustatory Deficits and Associations with Microbiota in Parkinson’s Disease. Int. J. Mol. Sci..

[6] Vascellari S., Manzin A. (2021). Parkinson’s disease: A prionopathy?. Int. J. Mol. Sci..

[7] Bluhm A., Schrempel S., von Hörsten S., Schulze A., Roßner S. (2021). Proteolytic α-Synuclein Cleavage in Health and Disease. Int. J. Mol. Sci..

[8] Di Martino R., Sisalli M.J., Sirabella R., Della Notte S., Annunziato L., Feliciello A., Scorziello A. (2021). NCX3-induced mitochondrial dysfunction in midbrain leads to neuroinflammation in striatum of A53T-α-synuclein transgenic old mice. Int. J. Mol. Sci..

[9] Cuenca-Bermejo L., Pizzichini E., Gonçalves V.C., Guillén-Díaz M., Aguilar-Moñino E., Sánchez-Rodrigo C., González-Cuello A.-M., Fernández-Villalba E., Herrero M.T. (2021). A New Tool to Study Parkinsonism in the Context of Aging: MPTP Intoxication in a Natural Model of Multimorbidity. Int. J. Mol. Sci..

[10] Ka Young Kim K.Y., Chang K.A. (2021). Therapeutic Potential of Magnetic Nanoparticle-Based Human Adipose-Derived Stem Cells in a Mouse Model of Parkinson’s Disease. Int. J. Mol. Sci..

[11] Fajardo-Serrano A., Rico A.J., Roda E., Honrubia A., Arrieta S., Goiaz Ariznabarreta G., Chocarro G., Lorenzo-Ramos E., Pejenaute A., Vázquez A. (2021). Adeno-Associated Viral Vectors as Versatile Tools for Parkinson’s Research, Both for Disease Modeling Purposes and for Therapeutic Uses. Int. J. Mol. Sci..

[12] Bonilauri A., Sangiuliano Intra F., Rossetto F., Borgnis F., Baselli G., Baglio F. (2022). Whole-Head Functional Near-Infrared Spectroscopy as an Ecological Monitoring Tool for Assessing Cortical Activity in Parkinson’s Disease Patients at Different Stages. Int. J. Mol. Sci..

[13] Tang C., Liu M., Zhou Z., Li H., Yang C., Yang L., Xiang J. (2023). Treadmill Exercise Alleviates Cognition Disorder by Activating the FNDC5: Dual Role of Integrin αV/β5 in Parkinson’s Disease. Int. J. Mol. Sci..

[14] Serra M., Pinna A., Costa G., Usiello A., Pasqualetti M., Avallone L., Morelli M., Napolitano F. (2021). Involvement of the Protein Ras Homolog Enriched in the Striatum, Rhes, in Dopaminergic Neurons’ Degeneration: Link to Parkinson’s Disease. Int. J. Mol. Sci..

